# Pathway crosstalk between the central metabolic and heme biosynthetic pathways in *Phanerochaete chrysosporium*

**DOI:** 10.1007/s00253-023-12846-0

**Published:** 2024-01-06

**Authors:** Daisuke Miura, Ryoga Tsurigami, Hiroyuki Kato, Hiroyuki Wariishi, Motoyuki Shimizu

**Affiliations:** 1 Biomedical Research Institute, National Institute of Advanced Industrial Science and Technology (AIST), Tsukuba, Ibaraki, 305-8566 Japan; 2 Faculty of Agriculture, Meijo University, Nagoya, Aichi 468-8502 Japan; 3 Faculty of Arts and Science, Kyushu University, Fukuoka, Fukuoka 819-0395 Japan

**Keywords:** Basidiomycete, Heme, Heme-binding protein, Pathway crosstalk

## Abstract

**Abstract:**

A comprehensive analysis to survey heme-binding proteins produced by the white-rot fungus *Phanerochaete chrysosporium* was achieved using a biotinylated heme–streptavidin beads system. Mitochondrial citrate synthase (PcCS), glyceraldehyde 3-phosphate dehydrogenase (PcGAPDH), and 2-Cys thioredoxin peroxidase (mammalian HBP23 homolog) were identified as putative heme-binding proteins. Among these, PcCS and PcGAPDH were further characterized using heterologously expressed recombinant proteins*.* Difference spectra of PcCS titrated with hemin exhibited an increase in the Soret absorbance at 414 nm, suggesting that the axial ligand of the heme is a His residue. The activity of PcCS was strongly inhibited by hemin with *Ki* oxaloacetate of 8.7 μM and *Ki* acetyl-CoA of 5.8 μM. Since the final step of heme biosynthesis occurred at the mitochondrial inner membrane, the inhibition of PcCS by heme is thought to be a physiological event. The inhibitory mode of the heme was similar to that of CoA analogues, suggesting that heme binds to PcCS at His^347^ at the AcCoA–CoA binding site, which was supported by the homology model of PcCS. PcGAPDH was also inhibited by heme, with a lower concentration than that for PcCS. This might be caused by the different location of these enzymes. From the integration of these phenomena, it was concluded that metabolic regulations by heme in the central metabolic and heme synthetic pathways occurred in the mitochondria and cytosol. This novel pathway crosstalk between the central metabolic and heme biosynthetic pathways, via a heme molecule, is important in regulating the metabolic balance (heme synthesis, ATP synthesis, flux balance of the tricarboxylic acid (TCA) cycle and cellular redox balance (NADPH production) during fungal aromatic degradation.

**Key points:**

*• A comprehensive survey of heme-binding proteins in P. chrysosporium was achieved.*

*• Several heme-binding proteins including CS and GAPDH were identified.*

*• A novel metabolic regulation by heme in the central metabolic pathways was found.*

**Supplementary Information:**

The online version contains supplementary material available at 10.1007/s00253-023-12846-0.

## Introduction

As a prosthetic group, heme (protoporphyrin IX–iron complex) plays crucial roles in a series of proteins: an electron transfer molecule in cytochromes, an oxygen carrier in globins, a sensor molecule in bacterial FixL and CooA, and a redox catalyst in catalase, peroxidase, and cytochrome P450 (P450) (Raab 2001; Gilles-Gonzalez et al. 1991; Shelver et al. 1997; Omura 2005). In hemoproteins, oxidation, coordination, and spin states of the heme iron are controlled by the microenvironment of the protein active pocket. A cooperative action of heme and protein (amino acid residues) creates a designated function. On the other hand, free heme molecules interact with a variety of proteins, regulating their function; thus, heme also acts as an exogenous regulator. Mammalian 5-aminolevurinate (ALA) synthase (ALAS), which catalyzes the first and rate-determining step of the heme biosynthetic pathway, is negatively regulated by heme during translation and transportation to mitochondria. In addition, the heme biosynthetic pathway is regulated by heme in a feedback manner via an inhibition of ALAS (Lathrop and Timko 1993; Hamilton et al. 1991; Whitinc and Granick 1976). Glyceraldehyde 3-phosphate dehydrogenase (GAPDH) of the malaria parasite *Plasmodium falciparum* is inhibited by heme with a *Ki* of 0.2 μM (Campanale et al. 2003). Inhibition of GAPDH is thought to contribute to cellular redox balance, as it drastically changes the carbon flux from glycolysis to the pentose phosphate pathway, by which NADPH is generated. The activity of the transcription factors, yeast Hap1 and mammalian Bach1, is also known to be regulated by heme (Zhang and Guarente 1995; Ogawa et al. 2001). Heme molecules play a role in catalytic function and cellular regulation. Furthermore, free heme is known to be highly toxic to the cell since it acts as a photosensitizer producing reactive oxygen species, and as a strong oxidizer causing oxidative stress. Therefore, intracellular heme concentration should be strictly controlled.

Basidiomycetes are eukaryotic microorganisms, and many of the organisms are known to degrade lignin, one of the most recalcitrant biomaterials in nature (Higuchi 1980; Gold and Alic 1993; Kirk and Farrell 1987). One of the most extensively studied basidiomycetes, *Phanerochaete chrysosporium*, secretes a large amount of lignin and manganese peroxidases, which are extracellular hemoproteins responsible for the initial attack on lignin (Gold et al. 1989; Wariishi et al. 1989; Wariishi 2000). Peroxidase-driven lignin degradation by a non-specific, one-electron oxidation mechanism gives rise to a wide variety of low-molecular–weight aromatic fragments (Wariishi et al. 1991; Hammel and Moen 1991), which are further degraded intracellularly by a series of oxidative enzymes such as dehydrogenases, P450s, and dioxygenases (Wariishi 2000; del Cerro et al. 2021; Kijpornyongpan et al. 2022; Kato et al. 2022; Suzuki et al. 2023). Previously, the whole-genome sequence of *P. chrysosporium* has been completed by the US DOE Joint Genome Institute (Martinez et al. 2004), from which 154 P450 genes have been annotated. Involvement of P450 in the fungal metabolism of a series of aromatic compounds has also been reported (Teramoto et al. 2004; Matsuzaki and Wariishi 2005; Syed and Yadav 2012; Lin et al. 2022; Fessner et al. 2021; Sakai et al. 2018). *P. chrysosporium* produces a large amount of extracellular peroxidases and an array of intracellular P450 molecular species during lignin degradation; therefore, the fungus has to produce a large amount of heme. However, growth is completely shut down by exogenous addition of hemin at sub-μM concentrations, strongly suggesting that *P. chrysosporium* possesses a well-regulated system for heme biosynthesis, and that *P. chrysosporium* may be a model organism for mining new information on endogenous heme dynamics.

In the present study, to survey heme-derived cellular regulatory mechanisms, a comprehensive analysis of intracellular heme-binding proteins of *P. chrysosporium* was carried out. A biotinylated heme–streptavidin bead system (Ishida et al. 2003) was utilized for functional proteomics.

## Materials and methods

### Organism and culture conditions

*P. chrysosporium* (ATCC 34541) was grown from conidial inocula at 37 °C in a stationary culture (20-ml medium in a 200-ml Erlenmeyer flask). The medium (pH 4.5) used in this study was a ligninolytic medium as previously described (Shimizu et al. 2005; Kirk et al. 1978), with 1% glucose as carbon source and 1.2 mM ammonium tartrate as nitrogen source.

### Chemicals

All the substrates utilized in the present study were of analytical grade. All other chemicals were of reagent grade. Deionized water was obtained from Milli Q system (Merck-Millipore, Billerica, MA, USA).

### Preparation of biotinylated heme

Biotinylated heme was synthesized and purified as previously described (Ishida et al. 2003). Purified biotinylated heme was detected as a single peak on HPLC analysis monitored at 400 nm. Matrix-assisted laser desorption time-of-flight mass spectrometry (MALDI-TOF-MS) analysis showed a molecular mass of 969.70, and the electron absorption spectrum was almost identical to that of hemin in dimethyl sulfoxide.

### Preparation of cell-free extracts

After 3-day incubation, fungal mycelia were separated from the culture medium by centrifugation (5,000 ***g***, 5 min), washed three times with ice-cold 10 mM phosphate buffer (pH 7.0), and immediately frozen in liquid nitrogen. The mycelia were crushed with a mortar and pestle, then 1-ml phosphate buffer (100 mM, pH 7.0) containing 1 mM ethylenediaminetetraacetic acid, 1 mM phenylmethylsulfonyl fluoride, 1 mM dithiothreitol and 10-μl protease inhibitor cocktail (Sigma-Aldrich, St. Louis, MO, USA) were added. The homogenate was centrifuged at 11,000 ***g*** at 4 °C for 45 min. The supernatant was concentrated by ultrafiltration to 1.0–2.5 mg protein/ml and used as a cell-free extract. Protein concentration was determined by the method of Bradford with bovine serum albumin (BSA) as a standard (Bradford 1976).

### Affinity co-precipitation of heme-binding proteins using biotinylated heme–streptavidin beads

Streptavidin magnetic beads (Thermo Fisher Scientific, Chicago, IL, USA) were pre-washed five times with washing buffer (20 mM Tris–HCl, pH 8.0, 500 mM NaCl, 0.5% Tween 20). Cell-free extracts (0.6–1.5 ml) containing 1.0–2.5 mg protein were preincubated with or without 0.5 mM hemin at 37 °C for 10 min. Then, 80 μM biotinylated heme was added and incubated at 37 °C for 10 min. After removal of the insoluble materials by centrifugation, the supernatants were transferred into the tube containing pre-washed streptavidin beads (1 mg), and incubated at 37 °C for 10 min with gentle shaking. The resultant protein/biotinylated heme/streptavidin beads complexes were corrected using magnet and washed five times with washing buffer. Proteins were eluted from the beads by 10 M imidazole (pH 8.0). After desalting by acetone precipitation, samples were dissolved in sodium dodecyl sulfate (SDS) sample buffer and separated using SDS-polyacrylamide gel electrophoresis (SDS-PAGE).

### SDS-PAGE and peptide mass fingerprinting analysis

Protein samples were separated by SDS-PAGE on 10 or 12.5% gels, stained with Sypro Red, and visualized using a fluorescence image analyzer (Molecular Imager Fx; Bio-Rad, Hercules, CA, USA). The bands whose intensities decreased upon the addition of free hemin before biotinylated heme addition were excised and in-gel-digested with 20 ng/μl modified trypsin (Promega, Madison, WI, USA), in a minimal volume of 100 mM ammonium bicarbonate. Resultant peptides were extracted with 80% acetonitrile containing 0.05% trifluoroacetic acid and desalted with ZipTips C_18_ (Merck-Millipore, Burlington, MA, USA) and analyzed using MALDI-TOF–MS to acquire peptide mass fingerprints (PMF). α-Cyano-4-hydroxycinnamic acid was used as matrix, and internal mass calibration was performed using bradykinin (904.45 Da) and adrenocorticotropic hormone (2465.75 Da). PMFs were searched against the in silico *P. chrysosporium* protein database, using the MASCOT search engine, and identified as previously described (Shimizu et al. 2005).

### Purification of mitochondrial citrate synthase from *P. chrysosporium*

Ammonium sulfate was added to cell-free extracts, and precipitates obtained at 40–60% saturation were dissolved in a minimal amount of 10 mM phosphate buffer (pH 7.0) and dialyzed overnight against the same buffer. Citrate synthase (CS) of *P. chrysosporium* (PcCS) was purified by ATP–agarose as reported for mammalian CS (Mukherjee and Srere 1976). After overnight dialysis, the enzyme solution was applied to an ATP–agarose column previously equilibrated with 10 mM phosphate buffer (pH 7.0). The column was washed with 200-ml 10 mM phosphate buffer, then bound proteins were eluted with 10 mM phosphate buffer (pH 7.0) containing 0.5 mM oxaloacetate (OAA) and 0.5 mM coenzyme A (CoA). The *N*-terminal amino acid sequence was determined as previously described (Edman 1956).

### Cloning and expression of the *PcCit1* and *PcGAPDH* genes by *Escherichia coli*

A full-length PcGAPDH cDNA and PcCS cDNA with the sequence for the N-terminal 19 amino acids deleted, which are the mitochondrial signal sequences, were amplified by PCR using KOD-plus DNA polymerase (Toyobo Co., Ltd.; Osaka, Japan). Gene-specific primer sets were designed using the genomic sequence of *P. chrysosporium* (https://mycocosm.jgi.doe.gov/Phchr4_2/Phchr4_2.home.html). Primers (5′-ATGCCGGTCAAAGCAGGAAT-3′) and (5′-AGGGCACCGTCGACCTTCGC-3′) were used for amplifying the gene coding PcGAPDH (6383341). Primers (5′-GGATCCTCAGTTCTTGTCCTTGAACA-3′ and 5′-CATATGCGCTCCCTCCGCTTCGCCTC-3′), which contained *Nde*1 and *Bam*H1 restriction sites, respectively, were used for amplifying the gene coding mature PcCS (6342608). PcGAPDH and PcCS genes were subcloned into the pET-30 vector containing the sequence for a 6×Histidine-tag (His-tag) and pET-23a (Novagen, Darmstadt, Germany), respectively. Plasmids were isolated and transformed into *E. coli* strain BL21(DE3)pLysS (Promega, Madison, WI, USA), and the transformants were incubated in TB medium at 30 °C. Expression of recombinant proteins was induced by the addition of 1 mM (PcGAPDH) or 0.4 mM (PcCS) IPTG (isopropyl-β-D-thiogalactopyranoside) and incubated overnight. Recombinant PcGAPDH was purified by Ni-NTA column chromatography, and the His-tag was removed by enterokinase. Recombinant PcCS (rPcCS) was purified as described above for the wild-type PcCS. The cDNA encoding PcGAPDH from *P. chrysosporium* (strain ATCC 34541) was assigned DDBJ accession number AB272086, and cDNA encoding PcCS was assigned accession number AB272085.

### GAPDH assay and steady-state kinetic analysis

GAPDH assay was performed as previously described (Campanale et al. 2003). For steady-state kinetic analysis, the reaction mixture (1 ml) contained 40 mM phosphate (pH 7.6), 5.2 μg recombinant PcGAPDH (rPcGAPDH), 40 mM triethanolamine, 5 mM NAD^+^, and 0.6–2 mM glyceraldehyde 3-phosphate (GAP3). Reactions were monitored by the increase in absorbance at 340 nm (ε 6220 M^–1^ cm^–1^) for 1 min, and initial velocities were determined. Inhibitory experiments were done in the presence of 0–2.5 μM hemin. To examine the effect of hemin on GAPDH kinetics, PcGAPDH was incubated with either buffer (control) or hemin for 5 min prior to initiating the reaction. Kinetic parameters were determined from Lineweaver–Burk plots, and *Ki* values were determined from the secondary plot of each slope of the Lineweaver–Burk plots. Immediately prior to use, hemin was dissolved at 10 mM in 0.1 M NaOH and diluted with 40 mM Tris–HCl (pH 7.5).

### Peroxidative activity of GAPDH–hemin complex

Peroxidative activity of the PcGAPDH–hemin complex was investigated by using 3, 3′, 5, 5′-tetramethylbenzidine (TMB) as oxidation substrate. Before the assay, 100 μM PcGAPDH was incubated with hemin at 1:1 or 1:2 stoichiometry for 30 min at 4 °C in the dark. Reaction mixtures (1 ml) contained 40 mM Tris–HCl (pH 7.5), 125 μM TMB, 100 μM hydrogen peroxide, and 1 μM PcGAPDH with hemin at 1:1 or 1:2 stoichiometry. As a control, 1 or 2 μM hemin without PcGAPDH was used. The reaction was monitored at 655 nm upon the formation of benzidine cation radical for 1 min.

### CS assay and steady-state kinetic analysis

CS was assayed by the 5, 5′-dithiobis-2-nitrobenzoic acid (DTNB) method, as described previously (Srere et al. 1963). The reaction mixture (1 ml) contained 0.2 mM OAA, 0.2 mM acetyl-CoA (AcCoA), and 0.2 mM DTNB in 40 mM Tris–HCl buffer (pH 8.5). CS catalyzes the reaction of OAA with AcCoA, generating citrate and CoA. Thus, reactions were monitored by the amount of released CoA, which was followed at 412 nm (ε 13 600 M^–1^cm^–1^) by the formation of 2-nitro-5-mercaptobenzoic acid, via the reaction of CoA with DTNB. Reactions were monitored for 1 min, and initial velocity was determined. For steady-state kinetic analysis, the reaction mixture (1 ml) contained 56 ng rPcCS and 0.12 mM DTNB in 40 mM Tris–HCl buffer (pH 8.5), with a fixed concentration of OAA at 20 μM and varied concentrations of AcCoA at 3–12 μM, or vice versa. Inhibition studies were carried out in the presence of 0–2.5 μM hemin as described above. Kinetic parameters were determined from Lineweaver–Burk plots, and *Ki* values were determined from the secondary plot of the slopes of the Lineweaver–Burk plots. Hemin and OAA solutions were prepared immediately prior to use.

Heme-derived time-dependent inhibition of PcCS was investigated by incubating PcCS with hemin for 30 min, before adding substrates and DTNB. Mixtures (1.0 ml) containing 0.56 μg rPcCS and 0–5.7 μM hemin in 40 mM Tris–HCl (pH 8.5) were incubated for 0 or 30 min at 4 °C, then 0.2 mM OAA, 0.2 mM AcCoA, and DTNB were added. Because of instability of PcCS in basic solution (pH 8.5), a 30% decrease in activity was observed in the controls (no hemin addition). Therefore, the data were expressed as relative values against the controls. When 5.7 μM hemin was used, citrate formed was determined by HPLC because of spectroscopic interference with the Soret absorption. All experiments were repeated at least three times, and standard deviations were within 5% in each plot.

### Spectroscopic characterization recombinant PcCS bound to heme

Spectroscopic observation of binding heme to proteins was investigated by difference spectra technique utilizing purified recombinant proteins. Recombinant proteins (1.5 μM) were dissolved to 40 mM Tris–HCl buffer (pH 7.5), and the solution was mixed with hemin. After 16 μM hemin addition, the difference spectrum was recorded between 300 and 700 nm. The reference cuvette contains the same solution with no protein. Resonance Raman spectra were measured by excitation at 413.1 nm. Prior to measurement, proteins and hemin (each 20 μM) were dissolved to 40 mM Tris–HCl buffer (pH 7.5), and then applied to G-25 gel filtration column to remove the unbound heme from the heme-protein complex. Pyridine hemochromogen method was used for determining the molar ratio of PcCS and hemin after gel filtration.

### Homology modeling

A homology model of the PcCS tertiary structure was constructed with the MOE program (Chemical Computing Group Inc., Montreal, Canada), according to the manufacturer’s instructions and as previously described (Rupasinghe et al. 2003), using chicken CS (PDB code: 1AL6) as a template structure. The truncated amino acid sequence with a deletion of 19 amino acids of the N-terminal mitochondrial signal from the full-length sequence was applied to the homology model program. The terminal chemical structure of N-hydroxyamidocarboxymethyldethia coenzyme A in that model was replaced with that of AcCoA. After construction of the initial homology model, further energy minimization was performed using the Amber10 force field within the MOE distribution, until the final energy gradient became < 0.01 kcal/mol Å.

## Results

### Affinity selection of heme-binding proteins

Hemin–agarose was generally used for purification or proteomic studies of heme-binding proteins (Campanale et al. 2003; Iwahara et al. 1995), but it showed several drawbacks, such as a non-specific interaction with agarose. Ishida et al. (2003) have reported a superior method for affinity selection of heme-binding proteins, which causes much less non-specific interactions, by exchanging a ligand-immobilizer (Ishida et al. 2003). Thus, the biotinylated heme–streptavidin system was applied for functional proteomics of fungal heme-binding proteins.

**Fig. 1:**
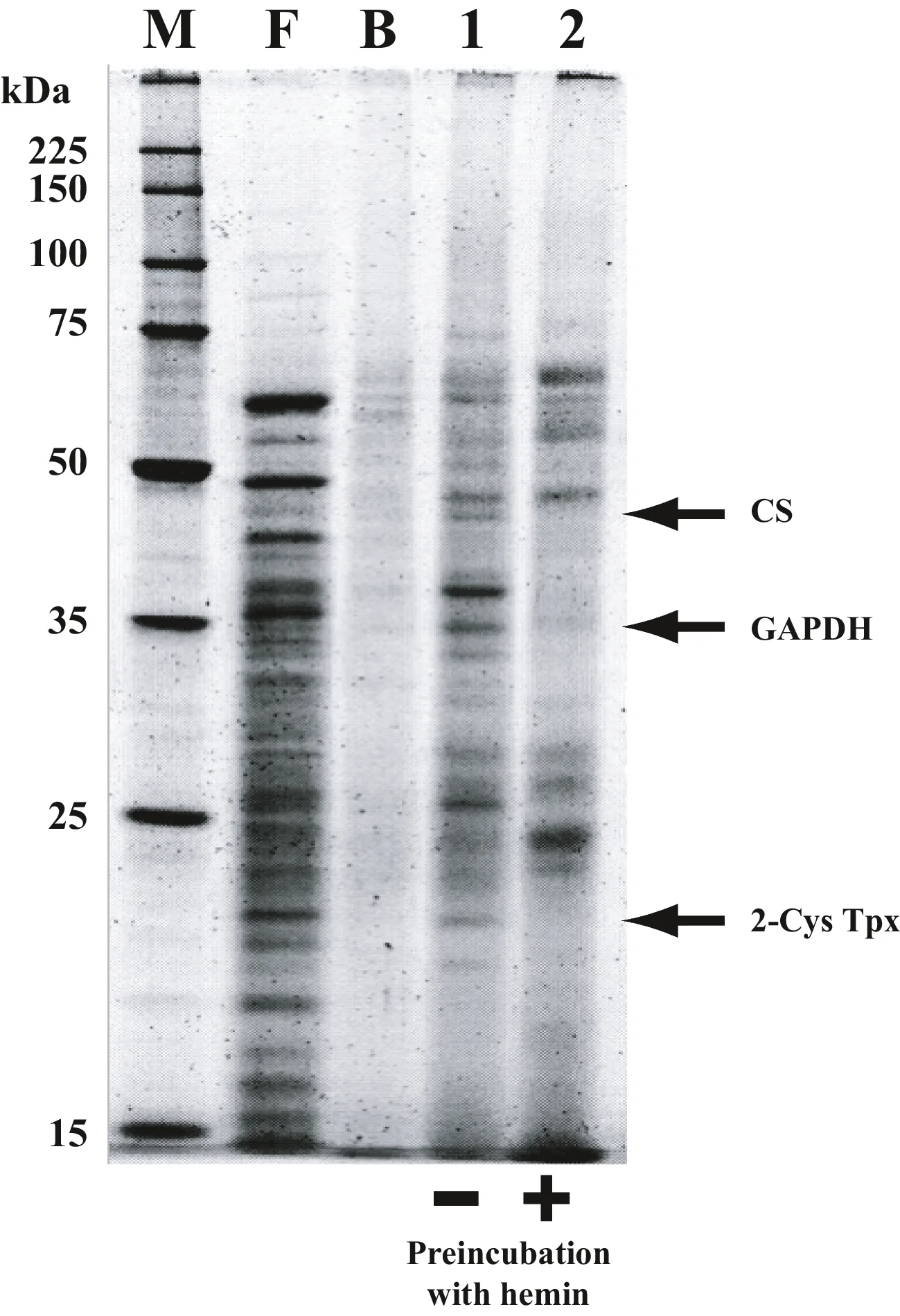
SDS-PAGE analysis of affinity-purified proteins acquired by using the biotinylated heme–streptavidin system. Affinity-purified proteins from *P. chrysosporium* utilizing biotinylated heme were resolved by SDS-PAGE. F, Flow-through elute of cell-free extracts loaded onto streptavidin beads. B, Imidazole elute fraction not incubated with biotinylated heme. Lane 1, imidazole elute fraction without hemin preincubation. Lane 2, imidazole elute fraction preincubated with 0.5 mM hemin before incubation with streptavidin beads. M, molecular markers. The bands specific for lane 1 other than CS and GAPDH have already been identified, but since these proteins were not metabolic enzymes, they are not mentioned in this paper

The main purpose of this study is to survey heme-binding protein under the ligninolytic condition in *P. chrysosporium*, because many kinds of hemoproteins such as peroxidases and P450s involved in the degradation of lignin and its-derived aromatic fragments were produced in the cell. We hypothesized that the production of a large amount of heme might trigger the expression of these heme-binding proteins. *P. chrysosporium* was incubated, and cell-free extracts were prepared. Synthesized biotinylated heme was added to cell-free extracts, resulting in the formation of protein–biotinylated heme complexes. The mixtures were then added to streptavidin beads, forming protein/biotinylated heme/streptavidin ternary complexes. Proteins were eluted from beads by 10 M imidazole, and then separated by SDS-PAGE (Fig. 1, lane 1). Preincubation of cell-free extracts with excess hemin before treatment with biotinylated heme decreased the number of bands observed (Fig. 1, lane 2). Protein bands, whose intensities decreased upon the addition of free hemin before the streptavidin bead treatment (observed in lane 1, but decreased or disappeared in lane 2), were isolated as candidate heme-binding proteins for further MS analysis. 2-Cys thioredoxin peroxidase, which was isolated as a candidate heme-binding protein (protein ID; 6378892), was a homolog of mammalian heme-binding protein 23 kDa (HBP23), confirming the validity of this method for surveying fungal heme-binding proteins (Fig. 1).

### GAPDH as a heme-binding protein

**Fig. 2:**
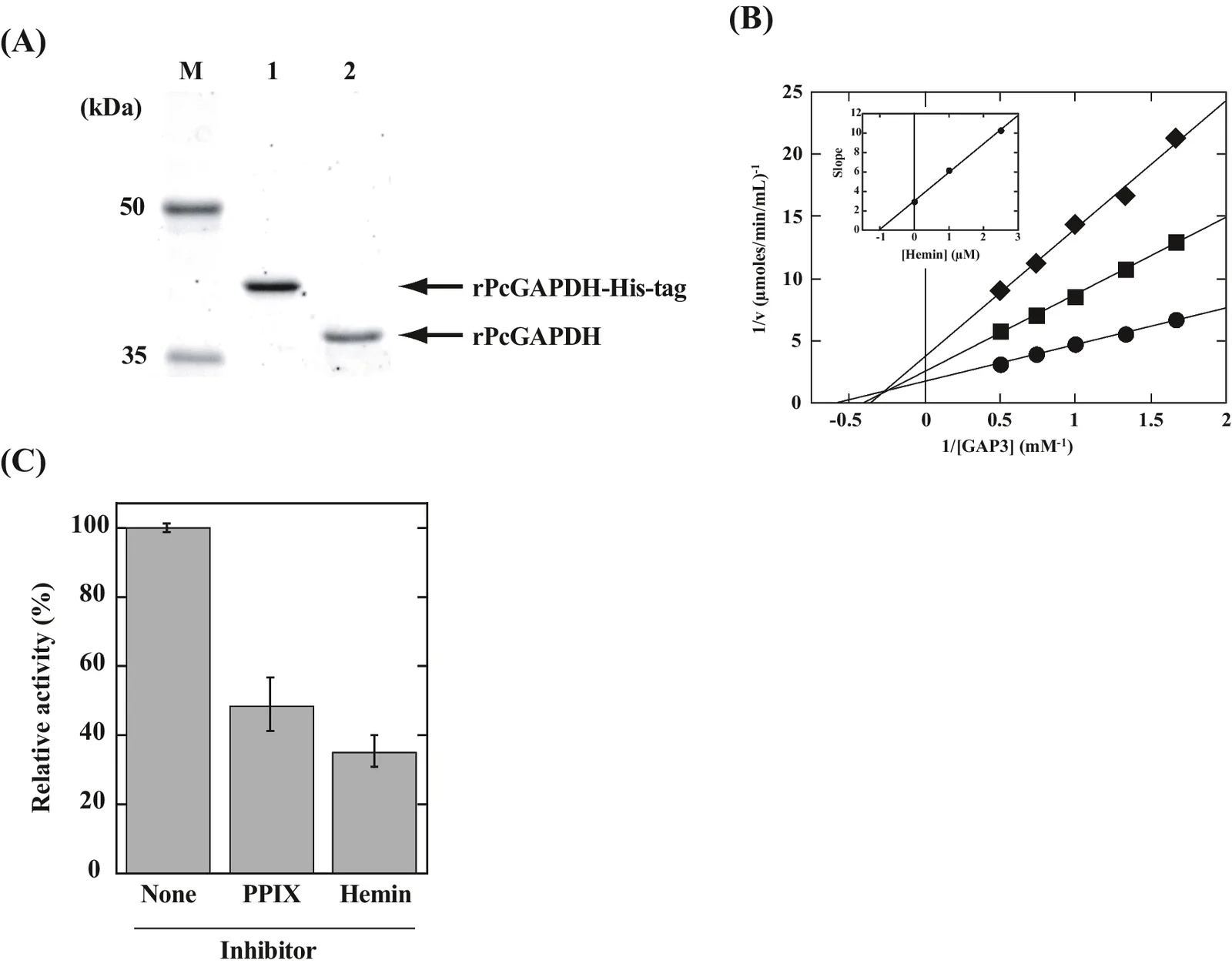
Heterologous expression of PcGAPDH and inhibitory effect of hemin on GAPDH reaction**. A** M, molecular weight marker; lane 1, rPcGAPDH-His-tag; lane 2, His-tag-cleaved rPcGAPDH. **B** Reaction mixtures containing 2.5 nM rPcGAPDH, 40 mM phosphate (pH 7.6), 40 mM triethanolamine, 5 mM NAD^+^, and 0.6–2 mM GAP3. The reciprocals of initial velocity were plotted against the reciprocals of variable substrate concentrations at fixed hemin concentrations of 0 (ℓ*●*), 1.0 (■) and 2.5 (◆) μM. *Inset*: secondary plots of each slope of Lineweaver–Burk plots against the concentration of hemin for determining inhibition constants. **C** Reaction mixtures containing 2.5 nM rPcGAPDH, 40 mM phosphate (pH 7.6), 40 mM triethanolamine, 5 mM NAD^+^, and 0.6 mM GAP3. Reactions were started by adding substrates followed by 5-min incubation with either buffer or 1 μM inhibitor (hemin or protoporphyrin IX, PPIX). Data indicate the relative value of GAPDH activity against that incubated with buffer. All experiments were performed in triplicate. Bars indicated as mean values ± standard deviation (error bars)

To confirm the effect of hemin on PcGAPDH (protein ID; 6383341), a recombinant protein was prepared (Fig. 2A). rPcGAPDH exhibited activity after removal of the His-tag. Fig. 2B clearly shows the effective inhibition of PcGAPDH by hemin. The inhibition constant was calculated from a secondary plot to be *K*_*i*_ = 1.0 μM (Fig. 2B, inset). PcGAPDH was also inhibited by protoporphyrin IX, as effectively as by hemin (Fig. 2C), indicating that the heme iron is not involved in the inhibitory interaction. Binding mode is still unclear, but it might not be due to interaction of the Cys residue at the active center and the heme iron. Furthermore, steady-state analysis indicated the occurrence of a mixed type of inhibitory pattern (Fig. 2B).

### Heterologous expression of a *CS* gene from *P. chrysosporium*

**Fig. 3:**
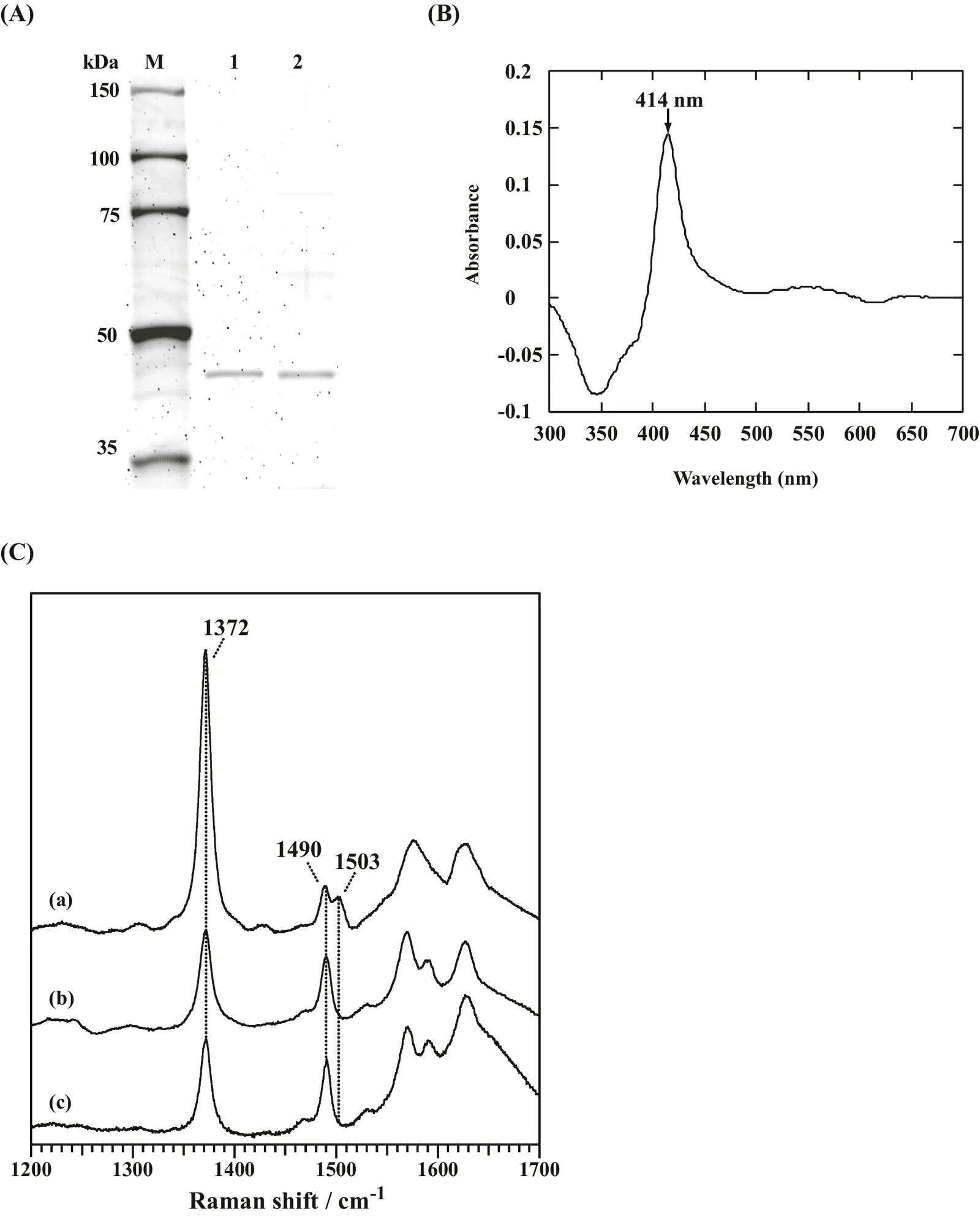
SDS-PAGE analysis of wild-type and rCS and spectroscopic characterization of heme–rPcCS complexes. **A** Lane 1, rPcCS was expressed using the pET expression system, as a mature peptide (N-terminal signal sequence removed), and purified by ammonium sulfate precipitation, DEAE–sepharose and ATP–agarose chromatography; lane 2, purified wtPcCS; M, molecular markers. **B** 1.5 μM rPcCS was mixed with 16 μM hemin in 40 mM phosphate buffer (pH 7.5), and the difference spectrum was recorded. Reference cuvettes contained the same solution with no protein. **C** Resonance Raman spectra were recorded with the 413.1 nm excitation. Samples (a, PcCS-heme complex; b, BSA-heme complex; c, hemin with no protein) were dissolved in 40 mM Tris–HCl at pH 7.5

CS is a key enzyme in the tricarboxylic acid (TCA) cycle and shown to be a dimeric enzyme in its active form (Remington et al. 1982). Mitochondrial PcCS (protein ID; 6342608) was identified as a heme-binding protein (Fig. 1). Furthermore, CS has been found to be upregulated by exogenously added aromatic compounds (Shimizu et al. 2005). To further examine the details, cDNA of *PcCit1* coding mitochondrial PcCS was cloned. Primers were designed from the sequence corresponding to the genome sequence. PSORT (http://psort.nibb.ac.jp/) and SignalP (http://www.cbs.dtu.dk/services/SignalP/) algorithms confirmed that PcCS is localized in mitochondria, and that the leader sequence might be cleaved between the nineteenth and twentieth residues. Wild-type PcCS (wtPcCS) was purified from cell lysates of *P. chrysosporium* by DEAE Sepharose and ATP–agarose column chromatography. The N-terminal sequence of purified wtPcCS was determined using a peptide sequencer, confirming that the cleavage site is between the nineteenth and twentieth residues. Then, cDNA coding mature PcCS (*PcCit1*) was cloned and heterologously expressed in *E. coli*. Active rPcCS was successfully purified by ATP–agarose. wtPcCS and rPcCS exhibited the same characteristics upon migration on SDS-PAGE (Fig. 3A) and PMFs (data not shown). The optimal pH for rPcCS was the same as that for wtPcCS, at 8.5. Steady-state kinetic analysis of rPcCS indicated *V*_*max* OAA_ = 31.4 nmoles/min/ml, *K*_*m* OAA_ = 6.2 μM against OAA, *V*_*max* AcCoA_ = 25.8 nmoles/min/ml, and *K*_*m* AcCoA_ = 4.2 μM against AcCoA. These kinetic parameters were almost identical to those of purified wtPcCS and similar to those of other fungal and mammalian CSs (Kispal and Srere 1991; Kurz et al. 1995; Ruijter et al. 2000; Matsuoka and Srere 1973).

### Spectral characteristics of heme–PcCS complex

**Fig. 4:**
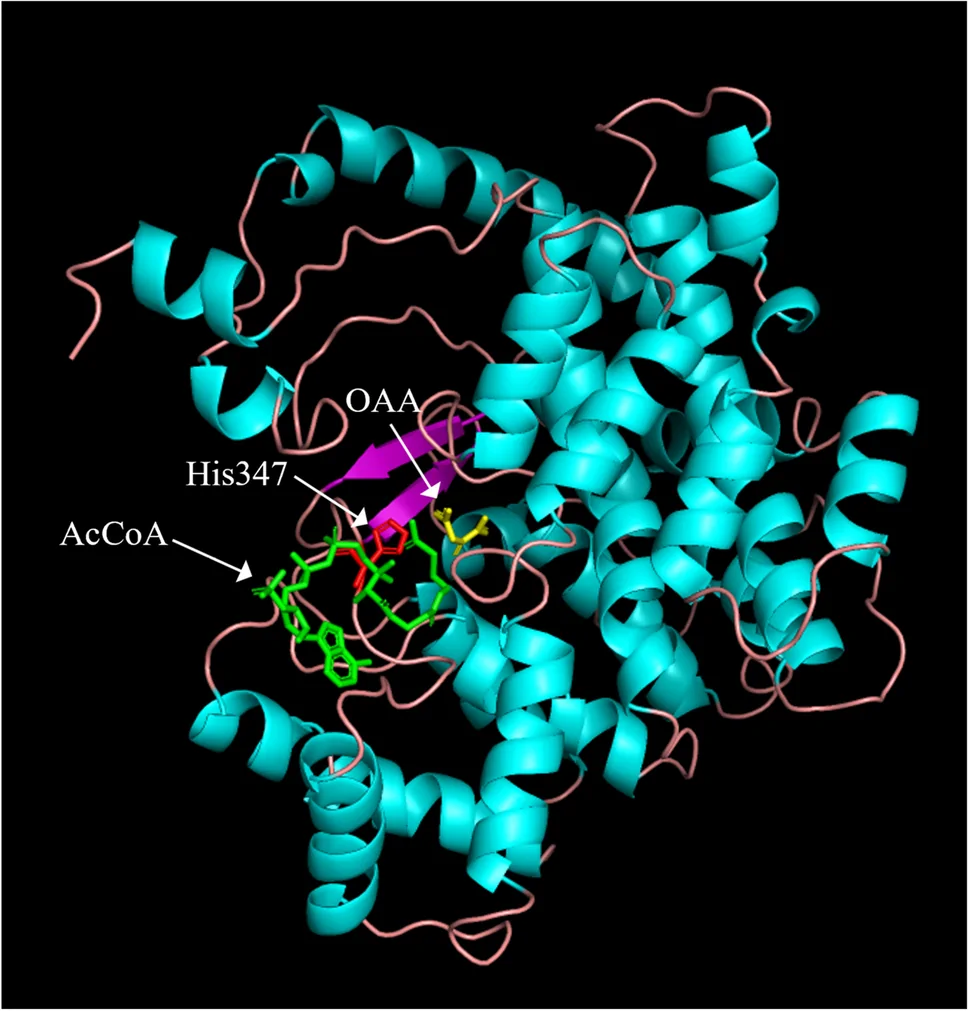
Putative heme-binding site of PcCS. The 3D structure of PcCS was constructed by homology modeling with the MOE program, using chicken CS (PDB code: 1AL6) as a template. Each substrate (OAA, yellow stick; AcCoA, green stick) and His residue (His^347^, red stick) were located at the catalytic center or surface of the protein. His^347^ (white arrow), which is at the AcCoA-binding site, was a putative heme-binding site of PcCS. PcCS has four His residues on the protein surface; however, three His residues except for His347 are not located near the active center, so they are omitted in this figure.

Fig. 3B shows the difference spectra of PcCS–ferric heme complexes. Absorption maximum and minimum were similar to those of HRI and HBP93 from rabbit sera (Uma et al. 2000; Tsutsui and Mueller 1982), rather than Hap1 and IRP2, strongly suggesting that the axial ligand of heme is a His residue. A resonance Raman spectrum of the PcCS-heme complex hexa-coodinate band was observed at 1503 cm^−1^ (Fig. 3C), and the PcCS-heme molar ratio was 2 that was determined by pyridine hemochromogen (data not shown). These data indicated that heme bound to PcCS at a histidine residue and made a bis-His type coordination with two CS protein and one heme molecule. Fig. 4 shows a 3D homology model of PcCS constructed from chicken CS (65% amino acid sequence identity to PcCS) as a template, indicating the existence of four His residues on the protein surface, with three (His^265^, His^301^, and His^347^) of these at the active site. These His residues are the candidate axial ligand for the heme binding site of PcCS.

### Inhibition of CS by heme

**Fig. 5:**
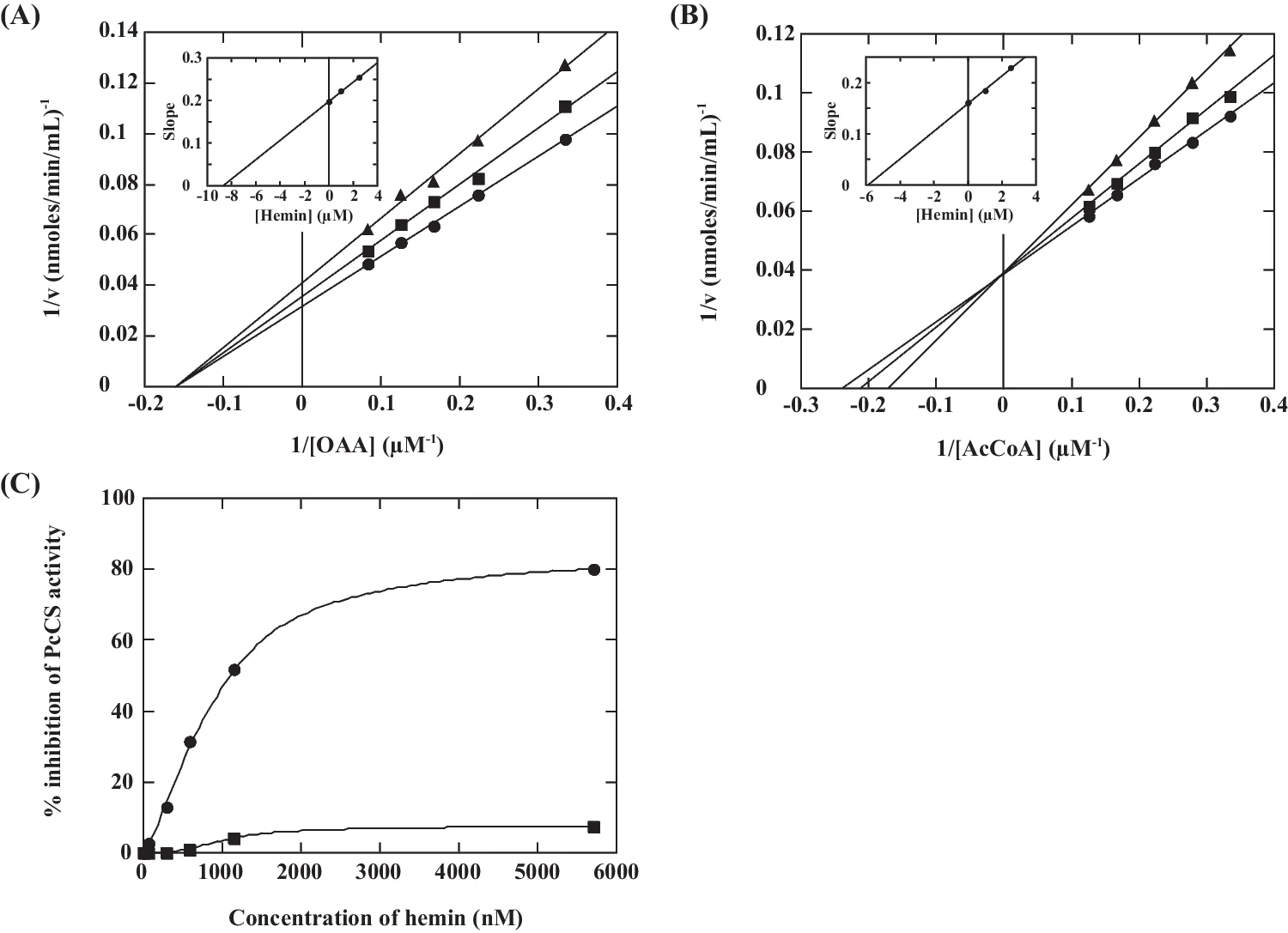
Inhibitory effect of hemin on PcCS reaction. A, B Steady-state kinetic analysis and inhibitory effect of heme for PcCS against A OAA and B AcCoA as variable substrates. Reaction mixtures contained 1.5 nM rPcCS, 40 mM Tris-HCl (pH 8.5), and 120 μM DTNB. Fixed substrate was added at 12 μM and variable substrate at 3–12 μM. The reciprocals of initial velocity were plotted against those of the variable substrate concentrations at fixed hemin concentrations of 0 (*ℓ●*), 1.0 (■), and 2.5 μM (_←_▲). Insets: secondary plots of each slope of Lineweaver–Burk plots against the concentration of hemin for determining inhibition constants. C Reaction mixtures containing 1.5 nM rPcCS, 40 mM Tris-HCl (pH 8.5), and 0–5.7 μM hemin were incubated for 0 (*ℓ■*) or 30 min (●) at 4 °C, then 0.2 mM OAA, 0.2 mM AcCoA and DTNB were added

Since heme binding to rPcCS was determined (Fig. 3B, C), the effect of hemin on the kinetic properties of rPcCS was examined. Double-reciprocal plots clearly indicated that hemin is an effective inhibitor for PcCS, and that hemin acts as a non-competitive inhibitor for OAA, and a competitive inhibitor for AcCoA (Fig. 5A, B). Secondary plots showed the inhibition constants of hemin as *K*_*i* OAA_ = 8.7 μM and *K*_*i* AcCoA_ = 5.8 μM, respectively (Fig. 5A, B, insets). This is the first report showing heme as an inhibitor of CS. It has been reported that CoA and propionyl-CoA act as competitive inhibitors of AcCoA and as non-competitive inhibitors of OAA for CS from rat kidney and *Drosophila melanogaster* (Matsuoka and Srere 1973; Lee et al. 1997). PcCS showed a high homology to rat CS (65% identity), suggesting that hemin binds at the AcCoA–CoA binding site of CS. Although several inhibitors have already been reported for other eukaryotic CSs, these inhibition constants are larger than 0.1 mM (Lee et al. 1997; Mahlen 1972; Porter and Wright 1977), indicating that hemin showed a 10-times higher inhibitory effect. Furthermore, protoporphyrin IX slightly inhibited PcCS at a concentration more than 10 times the inhibitory concentration of hemin (data not shown), suggesting that the binding and inhibition of heme to PcCS requires the presence of iron ions in the tetrapyrrol ring. The homology model indicated that the active site of PcCS contains three His residues, and that His^347^ is located at the AcCoA–CoA binding site (Fig. 4). The location of His^347^ on the surface of the protein allowed the hemin rapid access (Fig. 4).

One heme-responsive-motif (HRM) was found in PcCS, which could be a possible heme-binding site (Lathrop and Timko 1993; Zhang and Guarente 1995; Ishikawa et al. 2005; Yamanaka et al. 2003). From the alignment analysis, only certain fungi contain HRM (CP motif) in their CSs (Supplemental Fig. S1). No mammalian CS contains HRM (Supplemental Fig. S1). Therefore, the effect of hemin on pig CS (93% amino acid sequence identity to chicken CS) was examined, indicating that its activity was inhibited by hemin (Supplemental Fig. S2). Furthermore, the His residue corresponding to His^347^ in PcCS was conserved in either mammalian or fungal CSs. Combining all the data, the heme-binding site is concluded to be His^347^ of PcCS.

Malaria thioredoxin reductase has been reported to be inhibited by heme in a unique manner, with heme-induced inhibition occurring in either an immediate or time-dependent manner (Campanale et al. 2003). The above-mentioned kinetic data for PcCS indicate an immediate inhibitory effect. Therefore, heme-induced time-dependent inhibition of PcCS was examined by comparing the inhibitory effect immediately after and 30 min after hemin addition. Fig. 5C clearly shows the effect of 30-min incubation of PcCS with hemin on its enzymatic activity. With 30-min preincubation, the effect of hemin on PcCS activity was dramatically amplified, with a half-maximal inhibitory concentration (IC_50_) of 1 μM. PcCS is very sensitive to heme, and the effect of heme on PcCS is a physiological event. Furthermore, CS is located in the mitochondrial matrix (Robinson Jr and Srere 1985), and the enzymes involved in the first and final steps of heme biosynthesis, ALAS and ferrochelatase, are located at the mitochondrial inner membrane. Heme has been shown to negatively regulate the enzymatic activity of ALAS, and its translation and transportation to the mitochondria (Lathrop and Timko 1993; Hamilton et al. 1991; Whitinc and Granick 1976). From the deduced genomic sequence, *Phanerochaete* ALAS (protein ID; 6252801) was found to contain two HRMs (Cys^44^–Pro^45^ and Cys^84^–Pro^85^) at its N terminus; thus, it is likely that there is feedback inhibition of the heme biosynthetic pathway by heme in *P. chrysosporium*.

## Discussion

Many proteins have so far been reported as heme-binding proteins, and some of these have been reported to regulate their functional activities as a result of heme binding (Campanale et al. 2003; Zhang and Guarente 1995; Ogawa et al. 2001; Iwahara et al. 1995; Inuzuka et al. 2004; Sorgine et al. 2000; Uma et al. 2000; Tsutsui and Mueller 1982). Bach1 and Hap1, well-known heme-binding transcriptional factors in mammals and yeasts, respectively, regulate their transcriptional activity by binding to heme (Zhang and Guarente 1995; Ogawa et al. 2001). Binding of these transcription factors to heme occurs via a unique dipeptide (Cys and Pro) sequence called the HRM or CP motif. Indeed, many heme-binding proteins contain this short motif, and heme binding takes place at the Cys residue (Lathrop and Timko 1993; Zhang and Guarente 1995; Ishikawa et al. 2005). In this study, a comprehensive analysis to survey heme-binding proteins produced by the white-rot fungus *P. chrysosporium* was achieved using a biotinylated heme–streptavidin beads system. We successfully identified mitochondrial citrate synthase (PcCS), glyceraldehyde 3-phosphate dehydrogenase (PcGAPDH), and 2-Cys thioredoxin peroxidase (mammalian HBP23 homolog) as putative heme-binding proteins. Among them, enzymatic activity of PcGAPDH and PcCS was found to be strongly inhibited by heme.

GAPDH of the malaria parasite is reportedly inhibited by low concentrations of heme (Campanale et al. 2003). Inhibition of GAPDH in the glycolysis pathway causes a carbon flux shift to the pentose phosphate cycle, in which NADPH is generated. Since the malaria parasite is exposed to high oxidative stress by the large amount of heme from hemoglobin in infected erythrocytes, such a mechanism seems essential (Campanale et al. 2003). Biological significance of this phenomenon in this organism could be attributed to effective production of cellular reducing power by generating NADPH. Since *P. chrysosporium* is known to produce a large amount of heme proteins, the fungus faces a high level of oxidative stress. Thus, the interaction of heme with PcGAPDH may be attributed to an inhibitory effect.

Previously, a dramatic shift in the central metabolic pathway has been observed in *P. chrysosporium* (Shimizu et al. 2005). In glucose-supplemented medium, the fungus uses a unique “shortcut TCA/glyoxylate bicycle system” as a central energy metabolism pathway (Munir et al. 2001). In the shortcut TCA cycle of basidiomycetes, the steps from isocitrate to 2-oxoglutarate and then to succinyl-CoA are skipped, and succinate is directly formed from isocitrate by the action of isocitrate lyase, which shows much higher activity than isocitrate dehydrogenase. As a counterpart of succinate, glyoxylate is produced simultaneously. Interestingly, the general TCA cycle was activated in response to exogenously added aromatic compounds (Shimizu et al. 2005). This pathway switching has been shown to be responsible for (i) NADH generation for energy production by isocitrate and 2-oxoglutarate dehydrogenases and (ii) upregulation of enzymes such as ALAS, coproporphyrinogen oxidase, and uroporphyrinogen decarboxylase in the heme biosynthetic pathway branched at succinyl-CoA (Shimizu et al. 2005). Therefore, it is assumed that addition of exogenous aromatic compounds to *P. chrysosporium* causes a metabolic flux shift from a short-cut TCA/glyoxylate bicycle system to the general TCA cycle, which activates the heme biosynthetic pathway by providing succinyl-CoA for effective production of heme enzymes, such as lignin and manganese peroxidases and P450s. Upregulation of manganese peroxidase and P450s by exogenous aromatic compounds has also been reported (Shimizu et al. 2005; Li et al. 1995; Matsuzaki et al. 2008; Fessner et al. 2021). Over-produced heme may interact with PcGAPDH to stop the flux in glycolysis and set the flux into the pentose phosphate pathway for NADPH production. It is also worth mentioning that activation of the pentose phosphate pathway by adding aromatic compounds has also been observed at the proteome level (Shimizu et al. 2005).

Heme is coordinately bound to amino acid residues such as His and Cys in almost all hemoproteins. His is the axial ligand of heme in globins and peroxidases. Cys is found as the axial ligand in P450 and some sensor proteins (Raab 2001; Omura 2005). Heme-binding proteins have also been reported to bind to heme via a His residue in mammalian heme-regulated eIF2 kinase (HRI) (Inuzuka et al. 2004), and via Cys in ALAS, Hap1 and IRP2 (Lathrop and Timko 1993; Zhang and Guarente 1995; Ishikawa et al. 2005; Yamanaka et al. 2003). ALAS, Hap1 and IRP2 contain an HRM, and the Cys residue of HRM is known to be a heme axial ligand (Lathrop and Timko 1993; Zhang and Guarente 1995; Ishikawa et al. 2005; Yamanaka et al. 2003). It has been reported that the Soret peak appears at 362 nm upon binding ferric heme to HRM7 of Hap1 (Zhang and Guarente 1995), and at 370 nm upon binding the heme to HRM in the iron-dependent degradation domain of IRP2 (Ishikawa et al. 2005). On the other hand, heme binding to His^78^ and His^123^ in the N-terminal domain of HRI causes Soret absorption at 414 nm (Inuzuka et al. 2004; Uma et al. 2000).

**Scheme 1:**
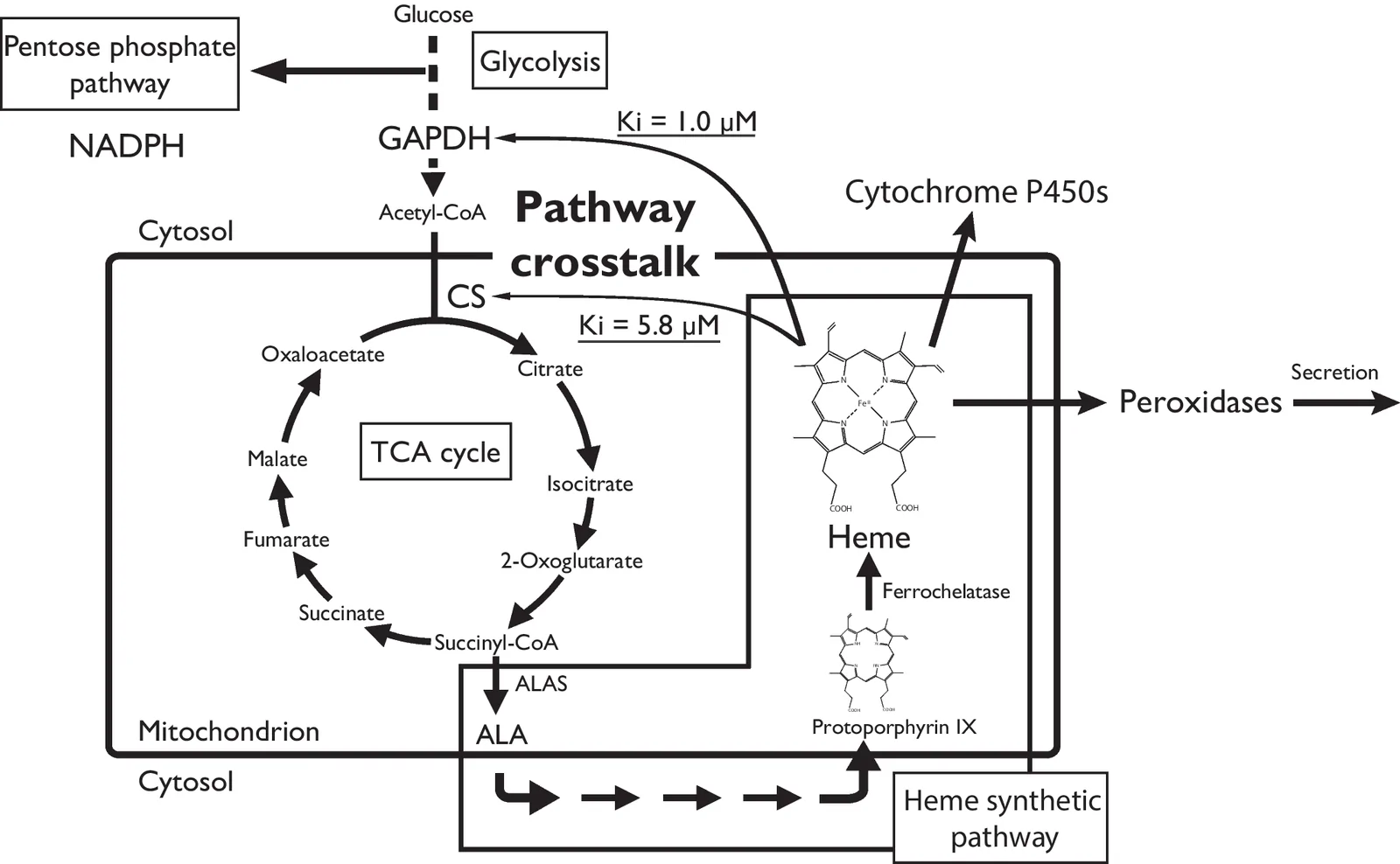
Pathway crosstalk between the central metabolic and heme synthetic pathways in *P. chrysosporium*

By exogenously adding 1.0 μM hemin to *P. chrysosporium* culture medium in the dark, growth was completely shut down (data not shown), suggesting the regulation for an intracellular amount of a free heme occurred at the synthetic level but not at the degradation level. Taking these observations together, a metabolic “pathway crosstalk” is proposed and summarized in Scheme 1. In *P. chrysosporium*, heme plays an important role as a prosthetic molecule for many cellular processes, in particular, ligninolytic enzymes. Upon addition of exogenous aromatic compounds, fungal cellular metabolism was dramatically shifted from basic energy metabolism to “defensive” or “productive” metabolism. Glucose uptake and consumption and NADPH and succinyl-CoA production were activated in response to exogenous aromatic compounds. Furthermore, the heme synthetic pathway was activated (Shimizu et al. 2005). Immediate activation of the heme synthesis against aromatics is crucial for the fungus to produce aromatics-degrading enzymes. On the other hand, free heme is highly toxic to the cell. Thus, metabolic overflow of heme biosynthesis causes highly stressful conditions in the mitochondria. The TCA cycle, including CS, is located in the mitochondrial matrix space (Mcalister-Henn and Small 1997). Ferrochelatase, the enzyme that catalyzes the final step of heme synthesis, is located at the mitochondrial inner membrane (Obi et al. 2022). It can be concluded that the inhibitory effect of heme on PcCS is a physiological event, and that PcCS is a primary target of heme. It is already established that CS is an important regulator of the TCA cycle and is affected by substrate concentration, and the redox and or energy state of the mitochondria in aerobic organisms (Srere 1975; Weitzman and Danson 1976). Therefore, regulation of PcCS activity by heme seems to buffer metabolic overflow, because the TCA cycle is a provider of succinyl-CoA, a precursor of heme.

**Fig. 6:**
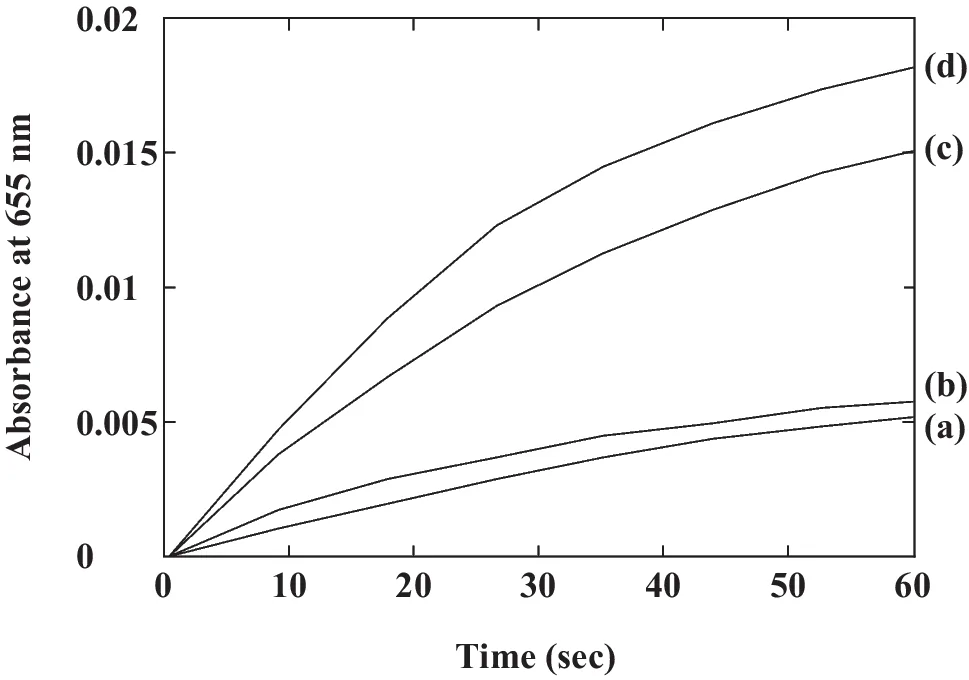
Peroxidative activity of GAPDH–hemin complex. Peroxidative activity of GAPDH–hemin complex was investigated by using TMB as an oxidation substrate. Reaction mixtures contained 40 mM Tris/HCl (pH 7.5), 125 μM TMB, 100 μM hydrogen peroxide, 1 μM PcGAPDH with hemin at 1:1 (a) or 1:2 (b) stoichiometry. As a control, 1 (c) or 2 (d) μM hemin without PcGAPDH was used. The reaction was monitored at 655 nm for 1 min

The inhibitory effect of heme on GAPDH in turn causes a metabolic shift to the pentose phosphate pathway, by suppressing the metabolic flow of glycolysis. Since lignin degradation by *P. chrysosporium* is an oxidative process, a large amount of reducing equivalents is required to maintain cellular redox balance. Additionally, glyoxal oxidase, which is a well-characterized extracellular H_2_O_2_-generating enzyme, is induced by vanillin (Shimizu et al. 2005). Antioxidative proteins such as superoxide dismutase, thioredoxin peroxidase, and hemoprotein catalase are also upregulated under the conditions (Shimizu et al. 2005). These indicate that the fungus faces oxidative stress under the ligninolytic conditions. It is well known that GAPDH is one of the most abundant proteins in the cytosol (Sirover 1999), so it was thought that PcGAPDH might bind free heme that is not utilized for production of hemoproteins, by making inactive complexes to avoid oxidative damage. In fact, the TMB-oxidizing ability of heme was suppressed by binding PcGAPDH in vitro (Fig. 6). Heme efflux from the mitochondrial matrix to the cytosol is required for hemoprotein production. However, the concentration of heme might be higher in the matrix. Thus, *Ki*_OAA_ values of 5.8 μM for PcCS and 1.0 μM for PcGAPDH are physiologically meaningful. Heme binding to PcCS and PcGAPDH seems to be essential for maintaining cellular metabolic and redox balance, as well as detoxification. This novel pathway crosstalk between the central metabolic and heme biosynthetic pathways, via heme, is also important for *P. chrysosporium* to optimize degradation pathways for a series of aromatic compounds.

This observation suggests that the heme-derived regulation of free heme concentration in the cytosol, and of the TCA metabolic flux, may occur in white-rot fungus *P. chrysosporium*, in which heme biosynthesis is initiated by ALAS on succinyl-CoA (C4 pathway).

## Supplementary information

ESM 1 (PDF 182 kb)

## Data Availability

All data generated or analyzed during this study are included in this published article and its supplementary information files.
