# Supplementary material for: Pathway crosstalk between the central metabolic and heme biosynthetic pathways in Phanerochaete chrysosporium
Source: Appl Microbiol Biotechnol. 2024 Jan 6;108:37. doi: 10.1007/s00253-023-12846-0 (PMC10771590; doi:10.1007/s00253-023-12846-0)
Supplement: Supplementary file 1 [file 253_2023_12846_MOESM1_ESM.pdf]

Applied Microbiology and Biotechnology

Supplementary materials

Pathway Crosstalk between the Central Metabolic and Heme Biosynthetic Pathways in *Phanerochaete chrysosporium*

Daisuke Miura[1]*, Ryoga Tsurigami[2], Hiroyuki Kato[2], Hiroyuki Wariishi[3] and Motoyuki Shimizu[2]*

[1] Biomedical Research Institute, National Institute of Advanced Industrial Science and Technology (AIST), Tsukuba, Ibaraki, 305-8566, Japan, [2] Faculty of Agriculture, Meijo University, Nagoya, Aichi 468-8502, Japan, [3] Faculty of Arts and Science, Kyushu University, Fukuoka, Fukuoka, 819-0395, Japan

Running Title: Metabolic pathway crosstalk in *P. chrysosporium.*

Keywords: Basidiomycete, heme, heme-binding protein, pathway crosstalk

*Corresponding Author:
Daisuke Miura; daisuke.miura@aist.go.jp
Motoyuki Shimizu; moshimi@meijo-u.ac.jp

```
PcCS    ------MSATVLRSLARQ-TP-RAAFGRSLRFASTGKQQNLKERLAELIPVELEHVKAVR    52
NcCS    MAPVMRLGSAALRSSIHLTSR-QTAFTAA--RCYSSKTQTLKERFAELLPENIEKIKALR    57
SsCS    ----MAL----LTAAARLFGAKNASCLVLAARHASASSTNLKDILADLIPKEQARIKTFR    52
HsCS    ----MAL----LTAAARLLGTKNASCLVLAARHASASSTNLKDILADLIPKEQARIKTFR    52
              :    * :  :     .::         :..  .**: :*:*:* :  ::*:.*

PcCS    AEHGKKAFGPVVVDQLYGGMRGLPALIWEGSVLDPEEGIRFRGKSIPECQELLPKAPGGN    112
NcCS    KEHGSKVVDKVTLDQVYGGARGIKCLVWEGSVLDAEEGIRFRGKTIPECQELLPKAPGGK    117
SsCS    QQHGNTVVGQITVDMMYGGMRGMKGLVYETSVLDPDEGIRFRGYSIPECQKMLPKAKGGE    112
HsCS    QQHGKTVVGQITVDMMYGGMRGMKGLVYETSVLDPDEGIRFRGFSIPECQKLLPKAKGGE    112
         :**..... :.:* :*** **:  *::* **** :******* :*****::**** **:

PcCS    EPLPEALFWLLVTGEVPTQEQVTALSKDWAARAAIPEFVEELLDRCPPTLHPMSQFSLAV    172
NcCS    EPLPEGLFWLLLTGEVPSEQQVRDLSAEWAARSDVPKFIEELIDRCPSDLHPMAQLSLAV    177
SsCS    EPLPEGLFWLLVTGQIPTEEQVSWLSKEWAKRAALPSHVVTMLDNFPTNLHPMSQLSAAI    172
HsCS    EPLPEGLFWLLVTGHIPTEEQVSWLSKEWAKRAALPSHVVTMLDNFPTNLHPMSQLSAAV    172
        *****.*****:**.:*:::**  ** :** *: :*..:  ::*. *  ****:*:* *:

PcCS    TALNHDSNFAKAYQQGISKKDYWGPVFEDCMDLIAKLPNIAGRIFRNVYGKGK-LPAIDA    231
NcCS    TALEHTSSFARAYAKGINKKEYWGYTFEDSMDLIAKLPTIAARIYQNVFKGGK-VAAVQK    236
SsCS    TALNSESNFARAYAEGIHRTKYWELIYEDCMDLIAKLPCVAAKIYRNLYREGSSIGAIDS    232
HsCS    TALNSESNFARAYAQGISRTKYWELIYEDSMDLIAKLPCVAAKIYRNLYREGSGIGAIDS    232
        ***:  *.**:** :** :..**   :**.******** :*.:*::*::  *. : *::

PcCS    NKDYSWNLATLLGFGENPAFVELMRLYITIHSDHEGGNVSAHTGKLVGSALSDPFLAFAA    291
NcCS    DKDYSFNFANQLGFGDNKDFVELLRLYLTIHTDHEGGNVSAHTTHLVGSALSSPFLSVAA    296
SsCS    KLDWSHNFTNMLGYTD-AQFTELMRLYLTIHSDHEGGNVSAHTSHLVGSALSDPYLSFAA    291
HsCS    NLDWSHNFTNMLGYTD-HQFTELTRLYLTIHSDHEGGNVSAHTSHLVGSAFSDPYLSFAA    291
        . *:* *::. **: :   *.** ***:***:*********** :*****:*.*:*:.**

PcCS    SLNGLAGPLHGLANQEVLIWLRRMQSKIGENASDEAVREYVWSTLKSGQVVPGYGHAVLR    351
NcCS    GLNGLAGPLHGLANQEVLNWLTEMKKVIGDDLSDEAITKYLWDTLNAGRVVPGYAHAVLR    356
SsCS    AMNGLAGPLHGLANQEVLVWLTQLQKEVGKDVSDEKLRDYIWNTLNSGRVVPGYGHAVLR    351
HsCS    AMNGLAGPLHGLANQEVLVWLTQLQKEVGKDVSDEKLRDYIWNTLNSGRVVPGYGHAVLR    351
        .:**************** ** .::. :*.: *** : .*:*.**::*:*****.*****

PcCS    KTDPRYTAQREFALKHLPNDPMFKLVGQIYNIVPGILLEAGKAKNPWPNVDAHSGVLLTH    411
NcCS    KTDPRYSAQRKFAQEHLPEDPMFQLVSQVYKIAPKVLTEHGKTKNPYPNVDAHSGVLLQH    416
SsCS    KTDPRYTCQREFALKHLPHDPMFKLVAQLYKIVPNVLLEQGKAKNPWPNVDAHSGVLLQY    411
HsCS    KTDPRYTCQREFALKHLPNDPMFKLVAQLYKIVPNVLLEQGKAKNPWPNVDAHSGVLLQY    411
        ******:.**:** :***.****:**.*:*:*.* :* * **:***:*********** :

PcCS    YGLTQMNFYTVLFGVSRAFGVAAQLIWDRALGAPLERPKSYSTGAIEKMFKDKN-         465
NcCS    YGLTEANYYTVLFGVSRAIGVLPQLIIDRAVGAPIERPKSYSTDKWIEICKKL--         469
SsCS    YGMTEMNYYTVLFGVSRALGVLAQLIWSRALGFPLERPKSMSTDGLIKLVDSK--         464
HsCS    YGMTEMNYYTVLFGVSRALGVLAQLIWSRALGFPLERPKSMSTEGLMKFVDSKSG         466
        **:*: *:*********:**  *** .**:* *:***** **    :: ..
```

Figure S1 Amino acid sequence alignment of CS isozymes and PcCS. Sequences of PcCS from *P. chrysosporium* (Protein ID; 6342608), NcCS from *Neurospora crassa* (Protein ID; P34085), SsCS from *Sus scrofa domesticus* (Protein ID; A0A0B8S099), and HsCS from *Homo sapiens* (Protein ID; O75390) are shown. The protein IDs of *Neurospora crassa*, *Sus scrofa domesticus*, and *Homo sapiens* were obtained from UniProt Knowledgebase (http://beta.uniprot.org) and the ID of *P. chrysosporium* was obtained from JGI Genome Portal (http://genome.jgi.doe.gov). The four His residues on the protein surface at the active site is boxed in red. Heme-responsive-motifs (HRM) conserved in fungal CS are bold and shaded in gray. The sequences were aligned using Clustal Omega program (http://www.clustal.org/omega/).

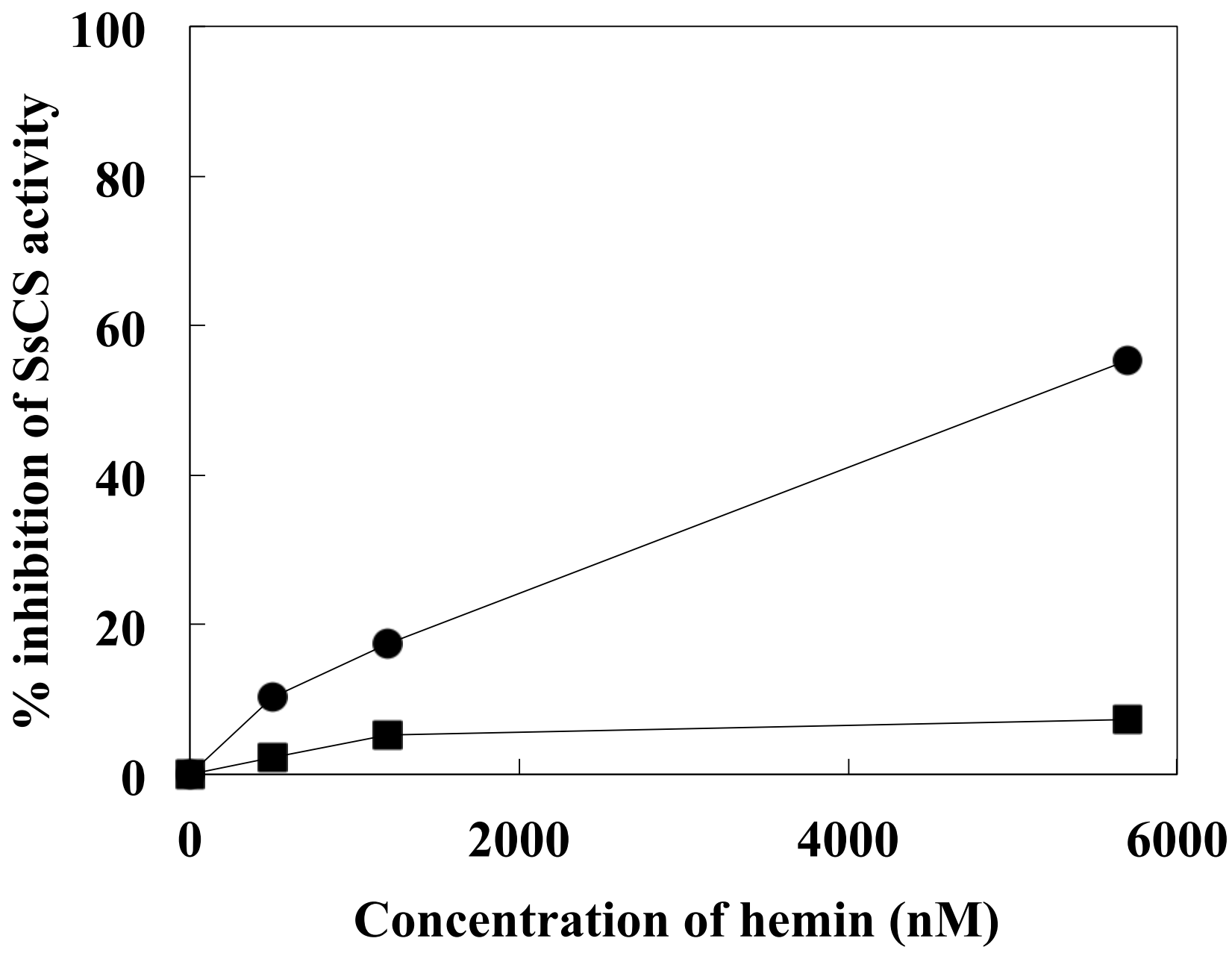

Figure S2 Inhibitory effect of hemin on SsCS reaction. Reaction mixtures containing 1.5 nM SsCS, 40 mM Tris/HCl (pH 8.5) and 0, 0.5, 1.2 and 5.7 μM hemin were incubated for 0 (■) or 60 min (●) at 4°C, then 0.2 mM OAA, 0.2 mM AcCoA, and DTNB were added.
