# Supplementary material for: Pathway crosstalk between the central metabolic and heme biosynthetic pathways in Phanerochaete chrysosporium
Source: Appl Microbiol Biotechnol. 2024 Jan 6;108(1):37. doi: 10.1007/s00253-023-12846-0 (PMC10771590; doi:10.1007/s00253-023-12846-0)
Supplement: Supplementary file 1 — (PDF 182 kb) [file 253_2023_12846_MOESM1_ESM.pdf]

Applied Microbiology and Biotechnology

Supplementary materials

Pathway Crosstalk between the Central Metabolic and Heme Biosynthetic Pathways in *Phanerochaete chrysosporium*

Daisuke Miura[1]*, Ryoga Tsurigami[2], Hiroyuki Kato[2], Hiroyuki Wariishi[3] and Motoyuki Shimizu[2]*

[1] Biomedical Research Institute, National Institute of Advanced Industrial Science and Technology (AIST), Tsukuba, Ibaraki, 305-8566, Japan, [2] Faculty of Agriculture, Meijo University, Nagoya, Aichi 468-8502, Japan, [3] Faculty of Arts and Science, Kyushu University, Fukuoka, Fukuoka, 819-0395, Japan

Running Title: Metabolic pathway crosstalk in *P. chrysosporium.*

Keywords: Basidiomycete, heme, heme-binding protein, pathway crosstalk

*Corresponding Author:
Daisuke Miura; daisuke.miura@aist.go.jp
Motoyuki Shimizu; moshimi@meijo-u.ac.jp

```
PcCS  ------MSATVLRSLARQ-TP-RAAFGRSLRFASTGKQQNLKERLAELIPVELEHVKAVR   52
NcCS  MAPVMRLGSAALRSSIHLTSR-QTAFTAA--RCYSSKTQTLKERFAELLPENIEKIKALR   57
SsCS  ----MAL----LTAAARLFGAKNASCLVLAARHASASSTNLKDILADLIPKEQARIKTFR   52
HsCS  ----MAL----LTAAARLLGTKNASCLVLAARHASASSTNLKDILADLIPKEQARIKTFR   52
            :    * :  :     .::         :..  .**: :*:*:* :  ::*:.*

PcCS  AEHGKKAFGPVVVDQLYGGMRGLPALIWEGSVLDPEEGIRFRGKSIPECQELLPKAPGGN   112
NcCS  KEHGSKVVDKVTLDQVYGGARGIKCLVWEGSVLDAEEGIRFRGKTIPECQELLPKAPGGK   117
SsCS  QQHGNTVVGQITVDMMYGGMRGMKGLVYETSVLDPDEGIRFRGYSIPECQKMLPKAKGGE   112
HsCS  QQHGKTVVGQITVDMMYGGMRGMKGLVYETSVLDPDEGIRFRGFSIPECQKLLPKAKGGE   112
       :**..... :.:* :*** **:  *::* **** :******* :*****::**** **:

PcCS  EPLPEALFWLLVTGEVPTQEQVTALSKDWAARAAIPEFVEELLDRCPPTLHPMSQFSLAV   172
NcCS  EPLPEGLFWLLLTGEVPSEQQVRDLSAEWAARSDVPKFIEELIDRCPSDLHPMAQLSLAV   177
SsCS  EPLPEGLFWLLVTGQIPTEEQVSWLSKEWAKRAALPSHVVTMLDNFPTNLHPMSQLSAAI   172
HsCS  EPLPEGLFWLLVTGHIPTEEQVSWLSKEWAKRAALPSHVVTMLDNFPTNLHPMSQLSAAV   172
      *****.*****:**.:*:::**  ** :** *: :*..:  ::*. *  ****:*:* *:

PcCS  TALNHDSNFAKAYQQGISKKDYWGPVFEDCMDLIAKLPNIAGRIFRNVYGKGK-LPAIDA   231
NcCS  TALEHTSSFARAYAKGINKKEYWGYTFEDSMDLIAKLPTIAARIYQNVFKGGK-VAAVQK   236
SsCS  TALNSESNFARAYAEGIHRTKYWELIYEDCMDLIAKLPCVAAKIYRNLYREGSSIGAIDS   232
HsCS  TALNSESNFARAYAQGISRTKYWELIYEDSMDLIAKLPCVAAKIYRNLYREGSGIGAIDS   232
      ***:  *.**:** :** :..**   :**.******** :*.:*::*::  *. : *::

PcCS  NKDYSWNLATLLGFGENPAFVELMRLYITIHSDHEGGNVSAHTGKLVGSALSDPFLAFAA   291
NcCS  DKDYSFNFANQLGFGDNKDFVELLRLYLTIHTDHEGGNVSAHTTHLVGSALSSPFLSVAA   296
SsCS  KLDWSHNFTNMLGYTD-AQFTELMRLYLTIHSDHEGGNVSAHTSHLVGSALSDPYLSFAA   291
HsCS  NLDWSHNFTNMLGYTD-HQFTELTRLYLTIHSDHEGGNVSAHTSHLVGSAFSDPYLSFAA   291
      . *:* *::. **: :   *.** ***:***:*********** :*****:*.*:*:.**

PcCS  SLNGLAGPLHGLANQEVLIWLRRMQSKIGENASDEAVREYVWSTLKSGQVVPGYGHAVLR   351
NcCS  GLNGLAGPLHGLANQEVLNWLTEMKKVIGDDLSDEAITKYLWDTLNAGRVVPGYAHAVLR   356
SsCS  AMNGLAGPLHGLANQEVLVWLTQLQKEVGKDVSDEKLRDYIWNTLNSGRVVPGYGHAVLR   351
HsCS  AMNGLAGPLHGLANQEVLVWLTQLQKEVGKDVSDEKLRDYIWNTLNSGRVVPGYGHAVLR   351
      .:**************** ** .::. :*.: *** : .*:*.**::*:*****.*****

PcCS  KTDPRYTAQREFALKHLPNDPMFKLVGQIYNIVPGILLEAGKAKNPWPNVDAHSGVLLTH   411
NcCS  KTDPRYSAQRKFAQEHLPEDPMFQLVSQVYKIAPKVLTEHGKTKNPYPNVDAHSGVLLQH   416
SsCS  KTDPRYTCQREFALKHLPHDPMFKLVAQLYKIVPNVLLEQGKAKNPWPNVDAHSGVLLQY   411
HsCS  KTDPRYTCQREFALKHLPNDPMFKLVAQLYKIVPNVLLEQGKAKNPWPNVDAHSGVLLQY   411
      ******:.**:** :***.****:**.*:*:*.* :* * **:***:*********** :

PcCS  YGLTQMNFYTVLFGVSRAFGVAAQLIWDRALGAPLERPKSYSTGAIEKMFKDKN-        465
NcCS  YGLTEANYYTVLFGVSRAIGVLPQLIIDRAVGAPIERPKSYSTDKWIEICKKL--        469
SsCS  YGMTEMNYYTVLFGVSRALGVLAQLIWSRALGFPLERPKSMSTDGLIKLVDSK--        464
HsCS  YGMTEMNYYTVLFGVSRALGVLAQLIWSRALGFPLERPKSMSTEGLMKFVDSKSG        466
      **:*: *:**********:**  *** .**:* *:***** **    :: ..
```

Figure S1 Amino acid sequence alignment of CS isozymes and PcCS. Sequences of PcCS from *P. chrysosporium* (Protein ID; 6342608), NcCS from *Neurospora crassa* (Protein ID; P34085), SsCS from *Sus scrofa domesticus* (Protein ID; A0A0B8S099), and HsCS from *Homo sapiens* (Protein ID; O75390) are shown. The protein IDs of *Neurospora crassa*, *Sus scrofa domesticus*, and *Homo sapiens* were obtained from UniProt Knowledgebase (http://beta.uniprot.org) and the ID of *P. chrysosporium* was obtained from JGI Genome Portal (http://genome.jgi.doe.gov). The four His residues on the protein surface at the active site is boxed in red. Heme-responsive-motifs (HRM) conserved in fungal CS are bold and shaded in gray. The sequences were aligned using Clustal Omega program (http://www.clustal.org/omega/).

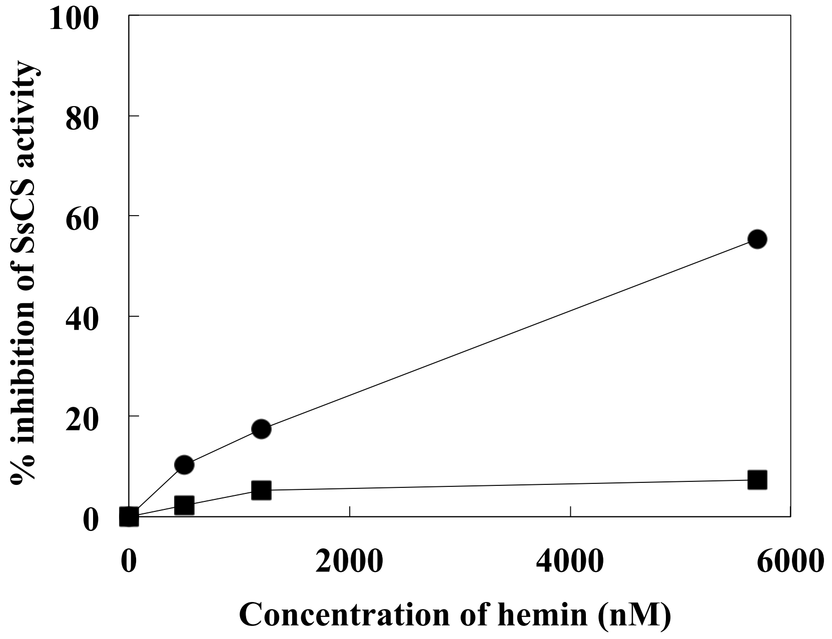

Figure S2 Inhibitory effect of hemin on SsCS reaction. Reaction mixtures containing 1.5 nM SsCS, 40 mM Tris/HCl (pH 8.5) and 0, 0.5, 1.2 and 5.7 μM hemin were incubated for 0 (■) or 60 min (●) at 4°C, then 0.2 mM OAA, 0.2 mM AcCoA, and DTNB were added.
